# Psychopathic traits linked to alterations in neural activity during personality judgments of self and others

**DOI:** 10.1016/j.nicl.2018.02.029

**Published:** 2018-02-28

**Authors:** Philip Deming, Carissa L. Philippi, Richard C. Wolf, Monika Dargis, Kent A. Kiehl, Michael Koenigs

**Affiliations:** a Department of Psychology, University of Wisconsin-Madison, 1202 West Johnson St., Madison, WI 53706, USA; b Department of Psychiatry, University of Wisconsin-Madison, 6001 Research Park Blvd., Madison, WI 53719, USA; c Department of Psychological Sciences, University of Missouri-St. Louis, 1 University Blvd., St. Louis, MO 63121, USA; d The Mind Research Network and Lovelace Biomedical, 1101 Yale Blvd. NE, Albuquerque, NM 87106, USA; e Department of Psychology, University of New Mexico, 1 University of New Mexico, Albuquerque, NM 87131, USA

**Keywords:** Psychopathy, fMRI, Social cognition, Self-referential processing, Emotion, Psychopathology

## Abstract

Psychopathic individuals are notorious for their grandiose sense of self-worth and disregard for the welfare of others. One potential psychological mechanism underlying these traits is the relative consideration of “self” versus “others”. Here we used task-based functional magnetic resonance imaging (fMRI) to identify neural responses during personality trait judgments about oneself and a familiar other in a sample of adult male incarcerated offenders (*n* = 57). Neural activity was regressed on two clusters of psychopathic traits: Factor 1 (e.g., egocentricity and lack of empathy) and Factor 2 (e.g., impulsivity and irresponsibility). Contrary to our hypotheses, Factor 1 scores were not significantly related to neural activity during self- or other-judgments. However, Factor 2 traits were associated with diminished activation to self-judgments, in relation to other-judgments, in bilateral posterior cingulate cortex and right temporoparietal junction. These findings highlight cortical regions associated with a dimension of social-affective cognition that may underlie psychopathic individuals' impulsive traits.

## Highlights

• fMRI study of social cognition in psychopathic criminals
• Task was judgment about personality traits referring to oneself or another person.
• Subset of psychopathic traits linked to posterior cingulate responses on the task
• This brain-behavior relationship may underlie psychopathic traits.

## 1 Introduction

Psychopathy is a significant predictor of violent crime and recidivism that is present in roughly a quarter of adult prison inmates (Hare, 2003; Harris et al., 1991). The core features of this personality disorder can be divided into two clusters of correlated traits: “Factor 1” interpersonal/affective traits (e.g., egocentricity, deceitfulness, and lack of empathy), and “Factor 2” lifestyle/antisocial traits (e.g., impulsivity, irresponsibility, and poor behavioral controls). Specifying the neuropsychological mechanisms underlying these clusters of traits could lead to more targeted and effective treatments for psychopathic criminals.

One potential mechanism related to the interpersonal and affective traits in psychopathy is the relative consideration of “self” versus “others”. A number of laboratory studies have linked psychopathy to reduced sensitivity to the welfare or distress of others. For example, psychopathic individuals exhibit reduced electrodermal responses to images of others in physical pain (House and Milligan, 1976; Pfabigan et al., 2015) and deficits in recognizing emotional facial expressions and vocalizations (Dawel et al., 2012). Similar anomalies have been found in adolescents with callous-unemotional traits, which resemble adult psychopathy (de Wied et al., 2012). Psychopathic criminals also behave more selfishly (rather than cooperatively or pro-socially) than non-psychopathic individuals in economic exchange games (Koenigs et al., 2010; Mokros et al., 2008). Together, these findings suggest a bias towards self-focus, and away from consideration of others, in psychopathy.

Recent investigations of the neural correlates of psychopathic traits also point to potential abnormalities in self/other-processing. Psychopathic individuals show reduced activation of visual cortices to emotional faces (Decety et al., 2014) and reduced activation of amygdala when imagining others in pain (Decety et al., 2013). Once again, similar patterns of neural activation to others' emotions have been observed in adolescents with callous-unemotional traits (Marsh et al., 2008; Richell et al., 2003).

Furthermore, several groups have found alterations in the default mode network (DMN), a set of brain regions implicated in self-related processing (Buckner et al., 2008), in relation to psychopathy (Freeman et al., 2015; Juarez et al., 2013; Philippi et al., 2015; Pujol et al., 2012). Psychopathic offenders display attenuated deactivation of DMN regions when switching attention from themselves to external task-related goals (Freeman et al., 2015). Psychopathic offenders also exhibit reduced functional connectivity between DMN regions at rest (Motzkin et al., 2011; Pujol et al., 2012). Medial prefrontal and parietal regions of the DMN overlap extensively with the network of brain regions activated by tasks involving evaluation of personality traits related to oneself and others (Heatherton et al., 2006; Kelley et al., 2002; Whitfield-Gabrieli et al., 2011). However, no study has directly examined whether psychopathy is associated with abnormal activity in this network of cortical midline structures during processing of self-relevant versus other-relevant personality traits.

To address this empirical gap, we employed a functional magnetic resonance imaging (fMRI) paradigm in a sample of criminal offenders to determine whether interpersonal and affective psychopathic traits are associated with altered neural activity during personality judgments about oneself versus a familiar other. We hypothesized that Factor 1 (interpersonal/affective) psychopathic traits would be associated with greater activity during self-focused judgments, relative to other-focused judgments, in cortical midline brain regions involved in social cognition.

## 2 Materials and methods

### 2.1 Participants

Adult male inmates were recruited from a medium-security correctional institution in Wisconsin. All participants met the following inclusion criteria: between the ages of 18 and 55; no history of psychosis, bipolar disorder, epilepsy or stroke; not currently using psychotropic medications; no history of head injury with loss of consciousness for >30 min; higher than fourth grade English reading level; intact auditory and visual capabilities; IQ > 70; and no MRI contraindications. *n* = 60 participants met inclusion criteria and completed the fMRI task. Prior to participation, all subjects provided informed consent.

### 2.2 Assessments

Psychopathy was measured with the Psychopathy Checklist-Revised (PCL-R) (Hare, 2003), which consists of a semi-structured interview and file review. The 20 PCL-R items were scored on a scale of 0–2, yielding total and Factor scores (Hare et al., 1990). Additionally, the 20 items were subdivided into the following “Facets”: interpersonal (Facet 1), affective (Facet 2), irresponsible lifestyle (Facet 3), and antisocial (Facet 4) (Hare and Neumann, 2005). Participants had a mean PCL-R total score of 22.9 (range: 8.4–37). Intra-class correlations of PCL-R total scores from the larger sample from which these participants were drawn show high inter-rater reliability (*n* = 129, ICC = 0.97).

Depression was assessed with the Beck Depression Inventory (BDI-II) (Beck et al., 1996), which consists of 21 symptoms rated on a four-point scale. Anxiety was assessed with the Welsh Anxiety Inventory (WAI) (Welsh, 1956), which consists of 39 statements rated as true or false. IQ was estimated from the Wechsler Adult Intelligence Scale 3rd Ed. (WAIS-III) (Wechsler, 1997) vocabulary and matrix reasoning scales. Substance use disorder diagnoses were determined using the Structured Clinical Interview for the DSM-IV (SCID-IV) (First et al., 2012). To minimize the number of covariates used in statistical models, a single dichotomous variable was calculated for substance use disorder (present or absent), based on whether participants met criteria for abuse or dependence on any substance (alcohol, cannabis, cocaine, opioids, stimulants, sedatives, or hallucinogens) (Korponay et al., 2016; Wolf et al., 2015).

### 2.3 Trait judgment task

During the fMRI task (Kelley et al., 2002), participants made yes/no judgments about trait adjectives in three conditions: Self (“Does the word describe you?”), Other (“Does the word describe your mother?”), and Case (“Is the word written in uppercase letters?”). A cue word (Self, Mother, or Case), presented with each adjective, signaled the target of the participant's judgment. A fixation cross was presented between trials (mean inter-trial interval 3.7 s, range 0.6–8.2 s). The task was split into two separate runs, with 45 trials in each run. The resulting 90 trials were split evenly among the three conditions and traits within each condition were matched for valence, average number of syllables, word length, and word frequency (each *p* > .7).

The participant's mother was chosen as the target of the other-judgment condition to keep familiarity with the “other” person consistent between participants. However, given the adverse personal experiences of many criminal offenders, the degree of familiarity with their mothers was expected to vary. Thus, prior to task administration, participants reported familiarity with their mothers, and were instructed to consider another primary caregiver if necessary.

### 2.4 fMRI acquisition

Imaging data were collected on prison grounds in the Mind Research Network's 1.5 T mobile imaging unit, using a 32-channel head coil. Multiband echo planar images (EPIs) were collected with the following parameters: TR = 350 ms, TE = 39.0 ms, flip angle = 38°, FOV = 248 × 248 mm, phase encoding direction = posterior to anterior, slice thickness = 3.50 mm, voxel size = 3.5 × 3.5 × 3.5 mm^3^, 48 slices per volume and a total of 1560 volumes. The shorter TR for EPIs was made possible by multiband imaging (Feinberg and Setsompop, 2013). High-resolution T1-weighted anatomical scans were collected for each subject (TR = 2400 ms, TE = 1.9 ms, flip angle = 8°, FOV = 256 × 256 mm^2^, slice thickness = 1.00 mm, voxel size = 1 × 1 × 1 mm^3^ and 176 interleaved sagittal slices).

### 2.5 Data analysis

fMRI data were processed using AFNI (16.0) (Cox, 1996). Geometric distortions due to field inhomogeneity in the EPI scans were corrected using two EPI spin-echo sequences (one in the anterior-to-posterior phase encoding direction; one in the posterior-to-anterior direction). The first twenty EPI volumes were removed from each run. The remaining volumes were slice-time corrected and motion corrected by rigid body alignment, using the twenty-first EPI acquisition as a reference. EPIs were also smoothed with a 6 mm full-width at half-maximum Gaussian kernel and scaled to a mean of 100. T1-weighted anatomical scans were skull-stripped and intensity-normalized. EPI volumes were aligned to the anatomical scans, and both were registered to MNI-152 template space. The two EPI runs were then concatenated and modeled with gamma variate hemodynamic response functions aligned to stimulus onset times. Button press times and residual head motion after volume correction were also modeled as regressors of no interest. Estimated motion parameters were not significantly correlated with PCL-R scores (*p* > .07). Using the resulting statistical maps, we performed general linear tests between the three conditions (Self, Other, and Case).

In a final set of analyses, PCL-R scores were regressed on the condition contrasts (Self > Other, Self > Case, Other > Case) in the whole-brain. See Supplemental Materials for exploratory region of interest analyses. Psychopathy-related hemodynamic responses were considered significant at *p*_FWE_ < .05 (cluster size > 10 voxels at uncorrected *p* < .002) (Cox et al., 2017). Monte Carlo simulations (3dFWHMx with the –ACF option and 3dClustSim in AFNI) determined the cluster extent threshold (Eklund et al., 2016). Given the significant correlation between the two PCL-R Factors (*r* = 0.67), each Factor model controlled for the other Factor, and each Facet model controlled for the other Facets. This was done to examine the unique variance associated with each component of the PCL-R, as there is evidence that Factor 1 traits show divergent relationships to external correlates when included in the same models as Factor 2 traits (Hicks and Patrick, 2006). Additionally, each model statistically controlled for age, race, substance use disorder diagnosis (SCID-IV), IQ (WAIS-III), depression (BDI-II), and anxiety (WAI). Table 1 displays correlations between PCL-R scores and the continuous covariates. Three participants were excluded from analysis (final *n* = 57 subjects) for the following reasons, respectively: improper phase encoding during the functional scans, excessive motion on >20% of EPI time points, and failure to respond on >20% of trials.

**Table 1:** Correlations between PCL-R scores and continuous covariates.

	(1)	(2)	(3)	(4)	(5)	(6)
(1) PCL-R Total	–					
(2) PCL-R Factor 1	0.86⁎⁎⁎	–				
(3) PCL-R Factor 2	0.93⁎⁎⁎	0.67⁎⁎⁎	–			
(4) Age	−0.15	−0.05	−0.27	–		
(5) IQ	−0.21	−0.15⁎⁎	−0.25	0.11	–	
(6) Anxiety	0.26⁎	0.15	0.27	−0.14	−0.08	–
(7) Depression	0.15	0.15	0.14	−0.14	−0.28⁎	0.62⁎⁎⁎

Anxiety was measured by Welsh Anxiety Index (WAI). Depression was measured by Beck Depression Inventory (BDI-II).

⁎ *p* < .05.

⁎⁎ *p* < .01.

⁎⁎⁎ *p* < .001.

In addition, behavioral data (number of “yes” responses on Self and Other trials) were entered into linear mixed effects models, using the same covariates as the fMRI models.

## 3 Results

**Fig. 1:**
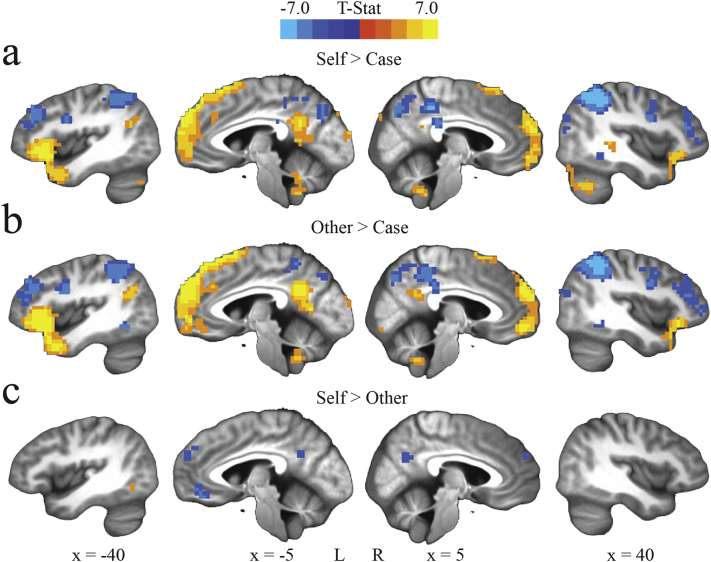
Areas of significant activation across the entire sample for a) the Self > Case contrast, b) the Other > Case contrast and c) the Self > Other contrast (uncorrected *p* = .002, *p*_FWE_ < .05).

Consistent with previous studies employing this fMRI task in normal adult subjects (Craik et al., 1999; Heatherton et al., 2006; Kelley et al., 2002), across our entire sample of inmates using whole-brain analyses (*p*_FWE_ < .05), we found that the Self > Case and Other > Case contrasts revealed a similar network of brain regions, which includes greater activity in medial prefrontal cortex (MPFC), posterior cingulate cortex (PCC)/precuneus, inferior frontal gyrus, and anterolateral temporal cortex, as well as reduced activity in dorsolateral prefrontal cortex and lateral parietal cortex (Fig. 1, Table 2). Several regions also showed preferential activity in the Other > Self contrast: PCC/precuneus, dorsomedial prefrontal cortex, bilateral anterior superior temporal sulcus, left temporoparietal junction (TPJ), and left posterior orbital gyrus.

**Table 2:** Regions showing significant differences between conditions in whole-brain analyses, regardless of PCL-R scores (uncorrected *p* = .002; *p*_FWE_ < .05).

Contrast	Brain region	Hemi.	Direction	Peak MNI coordinates			Size (voxels)
				X	Y	Z	
Self > Case							
Frontal	Medial prefrontal cortex	L/R	Pos	−5.2	−60.5	45.0	620
	Medial prefrontal cortex	L	Pos	−5.2	3.05	31.0	15
	Inferior frontal/anterolateral temporal cortex	L	Pos	−29.8	20.0	−11.0	488
	Inferior frontal/anterolateral temporal cortex	R	Pos	33.2	20.0	−14.5	192
	Middle frontal gyrus	R	Neg	43.8	34.0	20.5	98
	Middle frontal gyrus	L	Neg	−36.8	27.0	27.5	60
	Middle frontal gyrus	L	Neg	−29.8	−4.5	52.0	40
	Posterior superior frontal sulcus	R	Neg	26.2	−1.0	48.5	76
	Inferior frontal sulcus	R	Neg	47.2	2.5	24.0	67
	Inferior frontal sulcus	L	Neg	−43.8	2.5	24.0	26
	Orbital gyrus	L	Neg	−19.2	41.0	−4.0	20
Temporal	Posterior middle temporal gyrus	R	Neg	50.8	−43.0	−11.0	94
	Posterior middle temporal gyrus	L	Neg	−57.9	−50.0	−7.5	39
	Temporoparietal junction	L	Pos	−47.2	−60.5	27.5	56
	Parahippocampal gyrus	L	Pos	−22.8	−11.5	−11.0	15
Parietal	Inferior parietal cortex	L/R	Neg	33.2	−60.5	45.0	1264
	Posterior cingulate	L	Pos	−5.2	−50.0	24.0	134
	Posterior mid-cingulate cortex	L/R	Neg	5.2	−39.5	41.5	71
	Mid-cingulate cortex	L	Pos	−1.8	−11.5	34.5	11
Occipital	Occipital cortex	L	Pos	−8.8	−95.5	17.0	13
	Occipital cortex	R	Pos	8.8	−85.0	31.0	10
Cerebellum	Cerebellum	R	Pos	19.2	−74.5	−35.5	208
	Cerebellum	L	Pos	−19.2	−74.5	−35.5	70
	Cerebellum	L/R	Pos	5.2	−46.5	−35.5	55

Other > Case							
Frontal	Medial prefrontal cortex	L/R	Pos	−12.2	58.5	27.5	749
	Inferior frontal/anterolateral temporal cortex	L	Pos	−47.2	20.0	6.5	592
	Inferior frontal/anterolateral temporal cortex	R	Pos	36.8	27.0	−11.0	275
	Middle frontal gyrus	R	Neg	43.8	30.5	20.5	133
	Middle frontal gyrus	R	Neg	29.8	−4.5	45.0	76
	Middle frontal gyrus	L	Neg	−36.8	27.0	27.5	70
	Middle frontal gyrus	L	Neg	−29.8	−4.5	52.0	24
	Inferior frontal sulcus	L	Neg	−43.8	2.5	24.0	37
	Inferior frontal sulcus	R	Neg	47.2	2.5	27.5	86
Temporal	Temporoparietal junction	L	Pos	−47.2	−57.0	20.5	76
	Posterior middle temporal gyrus	L	Neg	−54.2	−50.0	−7.5	70
	Parahippocampal gyrus	L	Pos	−22.8	−11.5	−11.0	14
Parietal	Inferior parietal cortex	R	Neg	43.8	−46.5	45.0	850
	Inferior parietal cortex	L	Neg	−26.2	−67.5	31.0	564
	Posterior cingulate	L/R	Pos	−5.2	−50.0	27.5	189
	Posterior mid-cingulate cortex	L/R	Neg	8.8	−36.0	38.0	102
Occipital	Occipital cortex	R	Pos	8.8	−81.5	−7.5	36
	Occipital cortex	L	Pos	−8.8	−95.5	13.5	15
Cerebellum	Cerebellum	R	Pos	19.2	−74.5	−35.5	118
	Cerebellum	L	Pos	−26.2	−67.5	−32.0	41
	Cerebellum	L/R	Pos	−8.8	−50.0	−35.5	38

Self > Other							
Frontal	Dorsomedial prefrontal cortex	L/R	Neg	8.8	51.5	24.0	30
	Orbital gyrus	L	Neg	−5.2	37.5	−11.0	19
Temporal	Anterior superior temporal sulcus	L	Neg	−57.8	−4.5	−11.0	51
	Inferior temporal gyrus	L	Pos	−33.2	−64.0	−4.0	20
	Inferior temporal gyrus	R	Neg	19.2	−81.5	−7.5	11
Parietal	Posterior cingulate/Precuneus	L/R	Neg	1.8	−57.0	27.5	37

To test our main study hypothesis, we sought to identify brain regions where Factor 1 scores were associated with more activity when judging personality traits about oneself (“Self” condition) versus a familiar other (“Other” condition). Whole-brain analyses of the Self > Other contrast revealed no significant associations with Factor 1 scores (Table 3).

**Table 3:** Regions showing significant association between task contrasts and PCL-R scores in whole-brain analyses (uncorrected *p* = .002; *p*_FWE_ < .05).

Contrast	Brain region	Hemi.	Direction	Peak MNI coordinates			Size (voxels)
				X	Y	Z	
Self > Case							
PCL-R Total	None						
Factor 1	None						
Factor 2	None						
Facet 1	None						
Facet 2	None						
Facet 3	None						
Facet 4	None						

Other > Case							
PCL-R Total	None						
Factor 1	None						
Factor 2	None						
Facet 1	None						
Facet 2	None						
Facet 3	Medial prefrontal cortex	R	Pos	12.2	48.0	6.5	27
Facet 4	None						

Self > Other							
PCL-R Total	None						
Factor 1	None						
Factor 2	Temporoparietal junction	R	Neg	36.8	−57.0	13.5	17
	Posterior cingulate	R	Neg	12.2	−53.5	24.0	16
	Posterior cingulate	L	Neg	−1.8	−50.0	24.0	12
	Temporoparietal junction	R	Neg	36.8	−74.5	20.5	12
Facet 1	None						
Facet 2	None						
Facet 3	None						
Facet 4	None						

**Fig. 2:**
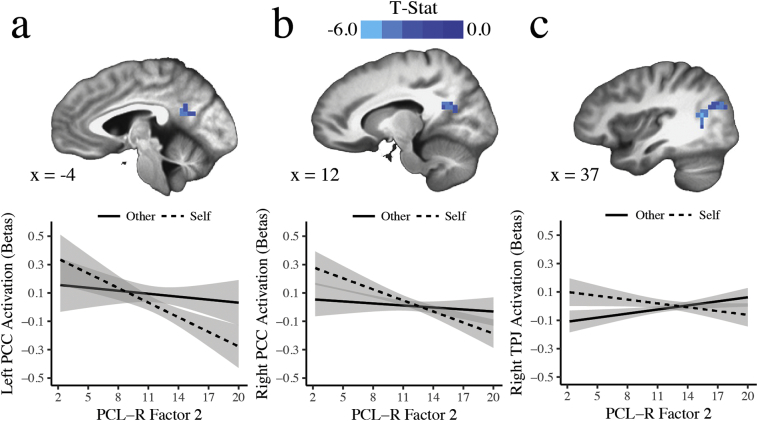
Regions significantly associated with PCL-R Factor 2 scores. Higher Factor 2 scores were related to diminished activity in a) left posterior cingulate cortex (PCC), b) right PCC and c) right temporoparietal junction (TPJ) during self-judgments, relative to other-judgments. Activation plots show regression lines with error bands representing 1 SE above and below the point estimate of the model.

Apart from the hypothesized results, the whole-brain analysis showed that Factor 2 scores were negatively related to Self > Other activation in the right and left PCC, and right TPJ (*p*_FWE_ < .05) (Fig. 2, Table 3). Results from analyses of PCL-R Total and Facet scores are displayed in Table 3.

Overall, participants were more likely to respond “yes” on Self trials than Other trials, *F*(1, 56.80) = 14.74, *p* < .001. However, this effect was unrelated to PCL-R Total scores, Factor scores or Facet scores, *p*s > .25.

## 4 Discussion

This study examined the neural regions subserving self-focused versus other-focused cognition in psychopathy. We hypothesized that PCL-R Factor 1 traits (e.g., egocentricity, lack of empathy) would be related to greater activity during self-judgment, relative to other-judgment, in cortical midline regions involved in social cognition. No brain regions showed the predicted relationship between Factor 1 traits and neural activity. Instead, a pattern of results emerged linking PCL-R Factor 2 traits (e.g., impulsivity, irresponsibility) to reduced neural activity to self-judgments, relative to other-judgments in bilateral PCC and right TPJ. We consider each of these findings in turn.

Factor 2 traits were related to attenuated PCC activity in the Self > Other contrast. This finding remained significant even when the *n* = 7 participants who thought of a different other (i.e., not their mother) were excluded from analysis, suggesting that familiarity to the target other did not substantially affect PCL-R results. The PCC is an integral node of the DMN (Fransson and Marrelec, 2008) and may underlie the function of “getting caught up in” self-referential thought (Brewer et al., 2013). Although the DMN may play a role in attention and cognition more broadly (Raichle, 2015), it has been reliably implicated in self-processing (Buckner et al., 2008). As Factor 2 includes traits such as impulsivity and poor behavioral controls, this finding may reflect a diminished capacity for self-reflection, resulting in behavior that lacks proper forethought (Philippi and Koenigs, 2014).

Similarly, right TPJ activity was negatively associated with Factor 2 traits during self-judgments, relative to other-judgments. Numerous studies have implicated the right TPJ in theory of mind, or thinking about another's thoughts and beliefs (Saxe and Kanwisher, 2003; Saxe and Wexler, 2005; Scholz et al., 2009). However, there is ongoing debate about the overlap between DMN, which is putatively involved primarily in self-processing, and regions involved in social cognition, especially TPJ (Amft et al., 2015; Mars et al., 2012a; Spreng et al., 2008). Furthermore, TPJ is a heterogeneous region, with different subregions likely contributing to distinct cognitive processes (Krall et al., 2015) and interacting with distinct neural networks (Mars et al., 2012b). More work is required to delineate the role of TPJ during self- and other-processing, and its relationship with the impulsive and antisocial traits of psychopathy.

It is noteworthy that the two PCL-R Factors showed markedly different relationships with neural activity during the trait judgment task. Specifically, Factor 1 was not related to neural activity during self- or other-judgments, whereas Factor 2 was related to decreased activity during self-judgments in the PCC and TPJ. These distinct relationships suggest that Factor 1 and Factor 2 traits, although highly correlated in terms of PCL-R scores, display clear dissociations at the neural level. This finding is consistent with recent neuroimaging studies of white matter integrity (Wolf et al., 2015), cortical functional connectivity (Contreras-Rodríguez et al., 2015; Philippi et al., 2015) and gray matter volume (Korponay et al., 2016).

Our initial hypothesis—that Factor 1 traits would relate to greater cortical midline activity during self-judgment than other-judgment—was not supported by the data. Given the body of literature linking Factor 1 traits of psychopathy to egocentric, callous behavior, it is important to consider the boundaries and limitations of the current study. Neural activity during social cognition is modulated by familiarity with the target person (Qin and Northoff, 2011). The current study contrasted self-judgments with judgments of a familiar other. Future studies may assess whether Factor 1 traits relate to decreased cortical midline activity during assessment of others who are less familiar than a primary caretaker. Alternatively, the personality trait judgment task may not sufficiently engage the cognitive processes of heightened self-focus that we hypothesize to underlie Factor 1 traits. Specifically, it has been suggested that personality trait judgment tasks likely engage controlled as opposed to automatic self-focused thought (Lemogne et al., 2009). These distinct subcomponents of self-processing may rely on different neural correlates (Lemogne et al., 2009; but see Moran et al., 2009). Perhaps a task involving automatic self-focus and greater self-interest, such as comparing oneself to others or competing with others in a reward task, would elicit regions of greater neural activity in relation to Factor 1 scores.

The behavioral component of this paradigm (yes/no response) was not itself a particularly sensitive measure of self/other processing. However, we believe our fMRI results make relevant predictions for more sensitive behavioral measures of self-processing. For example, we would expect the self-reference effect in memory tasks (i.e., better recall of self-relevant stimuli) to diminish with increasing Factor 2 scores (Symons and Johnson, 1997). As another example, we would expect self-reported interest in self-reflection to decrease with increasing Factor 2 scores (Trapnell and Campbell, 1999). Such findings would align with the current relationship between Factor 2 traits and decreased activity in self-processing regions during the personality trait judgment task.

In order to assess the sensitivity of the main analyses to covariates, we also performed the following supplementary analyses: models with each covariate separately removed, and models with the categorical substance use disorder variable replaced by a continuous measure from the Addiction Severity Index (ASI) (McLellan et al., 1992). For the latter analyses, years of regular use (defined by the ASI as three or more times per week for a period of at least one month) were summed for alcohol and other substances. Findings remained essentially the same for each of the supplementary analyses; in every case Factor 2 scores were inversely related to activity for the Self > Other contrast in PCC and right TPJ. Furthermore, of the four clusters significantly related to PCL-R Factor 2, all four remained significant at a more stringent threshold of *p*_FWE_ < .02, and the two largest clusters (in right PCC and right TPJ) were significant at *p*_FWE_ < .01.

In sum, we have identified brain regions where altered functioning may disrupt self-reflective judgment in inmates high in psychopathic traits such as impulsivity. These findings highlight a key dimension of social-affective cognition that may underlie the impulsive and irresponsible features of psychopathy.

## Appendix A Supplementary data

Supplemental Materials Image 1
