# Supplementary material for: Psychopathic traits linked to alterations in neural activity during personality judgments of self and others
Source: Neuroimage Clin. 2018 Feb 28;18:575–81. doi: 10.1016/j.nicl.2018.02.029 (PMC5964831; doi:10.1016/j.nicl.2018.02.029)
Supplement: Supplementary file 1 — Supplemental Materials [file mmc1.docx]

**SUPPLEMENTAL MATERIALS**

The following data are results from exploratory region of interest (ROI) analyses. We obtained ROIs from a meta-analysis of fMRI studies of trait judgment tasks: left and right medial prefrontal cortex (MPFC), left and right posterior cingulate cortex (PCC), left temporoparietal junction (TPJ), and left superior temporal sulcus (Denny, Kober, Wager, & Ochsner, 2012). Psychopathy-related hemodynamic responses were considered significant at *p_FWE_* < .05 (uncorrected *p* < .002) using the following cluster extent thresholds: left MPFC (3 voxels), right MPFC (3 voxels), left PCC (2 voxels, right PCC (2 voxels), left TPJ (2 voxels), and left STS (2 voxels).

No clusters showed the hypothesized relationship between PCL-R Factor 1 (interpersonal/affective) scores and activity in the Self > Other contrast (**Table S1**).

As in the whole-brain analyses, Factor 2 (irresponsible lifestyle/antisocial) was related to attenuated left PCC activation during self-judgments, relative to other-judgments (**Table S1**). A similar left PCC cluster showed the same relationship with Facet 3 (irresponsible lifestyle) traits.

**SUPPLEMENTAL TABLES**

| **Table S1**  Regions showing significant association between task contrasts and PCL-R scores in ROI analyses (uncorrected *p* = .002; *p_FWE_* < .05) | | | | | | | | | | | | | | |
| --- | --- | --- | --- | --- | --- | --- | --- | --- | --- | --- | --- | --- | --- | --- |
| Contrast | Brain Region | Hemi. | | Direction | | Peak MNI Coordinates | | | | | | Size (Voxels) | |  |
|  |  |  |  |  |  | x | | y | | z | |  |  |  |
| Self > Case |  |  | |  | |  | |  | |  | |  | |  |
| PCL-R Total | *None* |  | |  | |  | |  | |  | |  | |  |
| Factor 1 | *None* |  | |  | |  | |  | |  | |  | |  |
| Factor 2 | *None* |  | |  | |  | |  | |  | |  | |  |
| Facet 1 | *None* |  | |  | |  | |  | |  | |  | |  |
| Facet 2 | *None* |  | |  | |  | |  | |  | |  | |  |
| Facet 3 | MPFC | R | | Pos | | 8.8 | | 55.0 | | -0.5 | | 3 | |  |
| Facet 4 | *None* |  | |  | |  | |  | |  | |  | |  |
| Other > Case |  |  | |  | |  | |  | |  | |  | |  |
| PCL-R Total | *None* |  | |  | |  | |  | |  | |  | |  |
| Factor 1 | *None* |  | |  | |  | |  | |  | |  | |  |
| Factor 2 | *None* |  | |  | |  | |  | |  | |  | |  |
| Facet 1 | *None* |  | |  | |  | |  | |  | |  | |  |
| Facet 2 | *None* |  | |  | |  | |  | |  | |  | |  |
| Facet 3 | *None* |  | |  | |  | |  | |  | |  | |  |
| Facet 4 | *None* |  | |  | |  | |  | |  | |  | |  |
| Self > Other |  |  | |  | |  | |  | |  | |  | |  |
| PCL-R Total | *None* | |  | |  | |  | |  | |  | |  | |
| Factor 1 | *None* | |  | |  | |  | |  | |  | |  | |
| Factor 2 | PCC | | L | | Neg | | -8.8 | | -50.0 | | 20.5 | | 8 | |
| Facet 1 | *None* | |  | |  | |  | |  | |  | |  | |
| Facet 2 | *None* | |  | |  | |  | |  | |  | |  | |
| Facet 3 | PCC | | L | | Neg | | -1.8 | | -57.0 | | 24.0 | | 3 | |
| Facet 4 | *None* | |  | |  | |  | |  | |  | |  | |
| Regions of interest included left and right medial prefrontal cortex (MPFC), left and right posterior cingulate cortex (PCC), left temporoparietal junction (TPJ) and left superior temporal sulcus (STS). | | | | | | | | | | | | | | |
